# Generalizing the application of machine learning predictive models across different populations: does a model to predict the use of renal replacement therapy in critically ill COVID-19 patients apply to general intensive care unit patients?

**DOI:** 10.62675/2965-2774.20240285-en

**Published:** 2024-04-09

**Authors:** Allan Rodrigo Murrieta França, Julia Nunes Cantarino, Jorge Ibrain Figueira Salluh, Leonardo dos Santos Lourenço Bastos

**Affiliations:** 1 Universidade Federal do Rio de Janeiro Rio de Janeiro RJ Brazil Postgraduate Program of Internal Medicine, Universidade Federal do Rio de Janeiro - Rio de Janeiro (RJ), Brazil.; 2 Pontifícia Universidade Católica do Rio de Janeiro Department of Industrial Engineering Rio de Janeiro RJ Brazil Department of Industrial Engineering, Pontifícia Universidade Católica do Rio de Janeiro - Rio de Janeiro (RJ), Brazil.; 3 Instituto D'Or de Pesquisa e Ensino Rio de Janeiro RJ Brazil Postgraduate Program, Instituto D'Or de Pesquisa e Ensino - Rio de Janeiro (RJ), Brazil.

## TO THE EDITOR

The widespread use of machine learning has created the possibility of generating robust prediction models for individual patients; however, caution is needed in their use for heterogeneous critically ill populations.^(1)^ Recent literature has demonstrated major advances in the field of acute kidney injury prediction and the need for renal replacement therapy (RRT).^(2)^ In a large multicenter cohort, we evaluated how a previously published model^(3)^ that predicts the need for RRT in coronavirus disease 2019 (COVID-19) intensive care unit (ICU) patients would perform in a general ICU patient.

**Figure 1:**
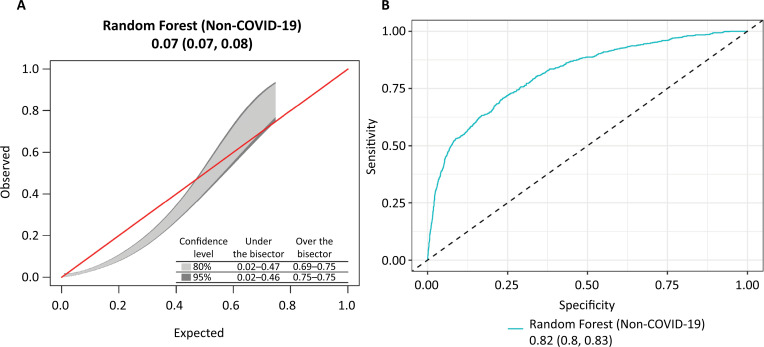
External validation results of calibration and discrimination for the final model.

Recently, using a data-driven methodology in a multicenter cohort of 14,374 critically ill COVID-19 patients, we developed and validated a machine learning prediction model to predict the use of RRT (the "COVID-19-RRT Model").^(3)^ In the present study, we performed an external validation of the "COVID-19-RRT Model" in a cohort of non-COVID-19 adult patients admitted to 126 ICUs in 2022 in a Brazilian private hospital network. The data were acquired using a solution used for quality assessment (Epimed Monitor).^(4)^ The study was approved by the Institutional Review Board after providing informed consent (*Instituto D'Or de Pesquisa e Ensino* [IDOR], CAAE:17079119.7.0000.5249). The prediction performance was evaluated in terms of calibration (plots and Brier's score) and discrimination (area under the ROC curve [AU-ROC]). A description of the materials and methods used are provided in the Supplementary Material (Table 1S, 2S and Figure 1S).

In 2022, 8,735 adult ICU patients without COVID-19 needed early respiratory support. Of these, 770 (8.8%) patients underwent RRT, a lower percentage than that in the development cohort (12%) (Table 1). Patients in the non-COVID-19 external validation cohort were older (median age 72 *versus* 56 years), more frequently female (54% *versus* 36%) and more frequently frail (43% *versus* 16%) than were those in the model development cohort. The median ICU stay was shorter (6 *versus* 10 days), and ICU mortality was lower compared to the development group (18% *versus* 22%). In the non-COVID-19 cohort, the model's AUC-ROC curve was 0.82 (95% confidence interval [95%CI]: 0.80 - 0.83), which was greater than that in the internal validation cohort (0.79; 95%CI: 0.78 - 0.82). Brier's score was comparable between the external validation dataset and the interval validation dataset; however, the calibration plots showed an overestimation of the predicted RRT probabilities, especially for patients at low risk (Figure 1).

The external validation dataset included 8,735 patients with general intensive care unit admissions who needed respiratory support upon admission (1st hour), 770 of whom needed renal replacement therapy in 2022. (A) Discrimination was evaluated using receiver operating characteristic curves and area under the curve curves. (B) The calibration belt evaluates the concordance between predicted and observed values: shaded areas represent the confidence bands (belt) of 80% and 95%, and the red line is the bisector line (observed = predicted). When the confidence bands significantly deviate from the bisector line, the corresponding quantiles of under- or overestimation are displayed in the lower right part of the plots. The p value represents a statistical test for the null hypothesis of calibration.

**Table 1:** Clinical characteristics and outcomes of critically ill general intensive care unit patients who needed respiratory support (within the first 24 hours after admission) and who received renal replacement therapy

Characteristic			n	Overall n = 8,735	Non-RRT n = 7,965	RRT n = 770
Age			8,735	72 (56 - 84)	72 (56 - 84)	71 (59 - 82)
Gender			8,735			
	Female			4,680 (54)	4,331 (54)	349 (45)
	Male			4,055 (46)	3,634 (46)	421 (55)
Charlson Comorbidity Index			8,659	1.00 (0.00 - 3.00)	1.00 (0.00 - 3.00)	3.00 (1.00 - 4.00)
Modified Frailty Index			8,735	2.00 (1.00 - 3.00)	2.00 (1.00 - 3.00)	2.00 (1.00 - 4.00)
Modified Frailty Index level			8,735			
	Frail			3,491 (40)	3,119 (39)	372 (48)
	Nonfrail			1,508 (17)	1,435 (18)	73 (9.5)
	Prefrail			3,736 (43)	3,411 (43)	325 (42)
Admission source			8,735			
	Emergency room			5,429 (62)	4,936 (62)	493 (64)
	Other unit at your hospital			743 (8.5)	631 (7.9)	112 (15)
	Outros			1,714 (20)	1,632 (20)	82 (11)
	Transfer from other hospital			324 (3.7)	283 (3.6)	41 (5.3)
	Ward/Floor			525 (6.0)	483 (6.1)	42 (5.5)
SAPS-3			8,735	54 (44 - 65)	54 (43 - 64)	65 (55 - 78)
SOFA score			6,823	2.0 (1.0 - 5.0)	2.0 (1.0 - 5.0)	7.0 (4.0 - 11.0)
Comorbidities			8,735			
	Hypertension		8,735	5,364 (61)	4,805 (60)	559 (73)
	Diabetes		8,735	2,909 (33)	2,584 (32)	325 (42)
	Obesity		8,735	508 (5.8)	468 (5.9)	40 (5.2)
	Immunosuppression		8,735	2,011 (23)	1,810 (23)	201 (26)
	Cardiovascular disease		8,735	2,998 (34)	2,661 (33)	337 (44)
	COPD or Asthma		8,735	1,510 (17)	1,385 (17)	125 (16)
	Malignancy		8,735	1,741 (20)	1,563 (20)	178 (23)
	Cerebrovascular disease		8,735	1,651 (19)	1,547 (19)	104 (14)
	Chronic Kidney disease		8,735	1,021 (12)	749 (9.4)	272 (35)
	Tobacco history		8,735	606 (6.9)	558 (7.0)	48 (6.2)
	Liver cirrhosis		8,735	197 (2.3)	158 (2.0)	39 (5.1)
	Other comorbidities		8,735	3,616 (41)	3,247 (41)	369 (48)
Physiology findings within the first hour						
	Lowest Glasgow Coma Scale (1 hour)		8,735	15.0 (11.0 - 15.0)	15.0 (12.0 - 15.0)	14.0 (6.0 - 15.0)
	Lowest platelets count (1 hour)		8,735	214 (161 - 274)	215 (163 - 275)	192 (130 - 263)
	Urea		8,735	44 (31 - 67)	42 (30 - 63)	75 (47 - 112)
	BUN		8,735	21 (14 - 31)	20 (14 - 29)	35 (22 - 52)
	Highest creatinine (1 hour)		8,735	0.94 (0.70 -1.36)	0.90 (0.70 -1.27)	1.80 (1.10 - 3.20)
Support at admission (1 hour)						
	Noninvasive ventilation		8,735	4,867 (56)	4,575 (57)	292 (38)
	Mechanical ventilation		8,735	2,300 (26)	1,981 (25)	319 (41)
	Vasopressor		8,735	2,099 (24)	1,747 (22)	352 (46)
Support during hospitalization						
	Noninvasive ventilation support		8,735	6,385 (73)	5,941 (75)	444 (58)
	High-flow nasal cannula		8,735	247 (2.8)	212 (2.7)	35 (4.5)
	Mechanical ventilation		8,735	3,130 (36)	2,582 (32)	548 (71)
		Days on mechanical ventilation	3,130	4 (1 - 12)	3 (1 - 10)	9 (3 - 18)
	ECMO		8,735	25 (0.3)	8 (0.1)	17 (2.2)
	Vasopressors		8,735	3,213 (37)	2,625 (33)	588 (76)
Outcomes						
	ICU mortality		8,735	1,607 (18)	1,244 (16)	363 (47)
	In-hospital mortality		8,735	2,064 (24)	1,623 (20)	441 (57)
	ICU length of stay		8,735	6 (3 - 13)	6 (3 - 11)	14 (6 - 29)
	Hospital length of stay		8,735	10 (5 - 20)	10 (5 - 19)	18 (8 - 38)

RRT - renal replacement therapy; SAPS-3 - Simplified Acute Physiology Score 3; SOFA - Sequential Organ Failure Assessment; COPD - chronic obstructive pulmonary disease; BUN - blood urea nitrogen; ECMO - extracorporeal membrane oxygenation; ICU - intensive care unit. The results are expressed as medians (interquartile ranges) or n (%).

Despite the good discrimination, the COVID-19-RRT Model overestimated the probability of needing RRT, especially in the "low-risk" strata.^(5)^ This may be explained by differences in the baseline severity of illness between COVID-19 patients and general ICU patients: the former had a lower severity at baseline, but the proportion of RRT use was greater than that in general ICU patients. Otherwise, a general ICU patient with a low disease severity at baseline will seldom require RRT. Therefore, despite good general performance, this model has limited clinical use for a mixed ICU population. Our study supports the need for models with better generalizability for the prediction of RRT and acute kidney injury in mixed ICU populations. Moreover, these findings should be interpreted with caution when translating the use of models developed for a specific population to a general group of critically ill patients.

## Data Availability

The data supporting this study's findings are available from the corresponding author upon reasonable request.

## References

[1] Huang CY, Grandas FG, Flechet M, Meyfroidt G (2020). Clinical prediction models for acute kidney injury in the intensive care unit: A systematic review. Rev Bras Ter Intensiva.

[2] Ramos FJ, França AM, Salluh JI (2022). Subphenotyping of critical illness: where protocolized and personalized intensive care medicine meet. Rev Bras Ter Intensiva.

[3] França AR, Rocha E, Bastos LS, Bozza FA, Kurtz P, Maccariello E (2023). Development and validation of a machine learning model to predict the use of renal replacement therapy in 14,374 patients with COVID-19. J Crit Care.

[4] Zampieri FG, Soares M, Borges LP, Salluh JI, Ranzani OT (2017). The Epimed Monitor ICU Database®: A cloud-based national registry for adult intensive care unit patients in Brazil. Rev Bras Ter Intensiva.

[5] Kurtz P, Bastos LS, Dantas LF, Zampieri FG, Soares M, Hamacher S (2021). Evolving changes in mortality of 13,301 critically ill adult patients with COVID-19 over 8 months. Intensive Care Med.

