# Supplementary material for: Generalizing the application of machine learning predictive models across different populations: does a model to predict the use of renal replacement therapy in critically ill COVID-19 patients apply to general intensive care unit patients?
Source: Crit Care Sci. 2024 Apr 9;36:e20240285en. doi: 10.62675/2965-2774.20240285-en (PMC11098073; doi:10.62675/2965-2774.20240285-en)
Supplement: Translation

# Translation: Generalização da aplicação de modelos preditivos de aprendizado de máquina em diferentes populações: um modelo que prevê o uso de terapia de substituição renal em pacientes com COVID-19 em estado grave se aplica a pacientes de unidades de terapia intensiva em geral?

**DOI:** 10.62675/2965-2774.20240285-pt

**Authors:** Allan Rodrigo Murrieta França, Julia Nunes Cantarino, Jorge Ibrain Figueira Salluh, Leonardo dos Santos Lourenço Bastos

**Affiliations:** 1 Rio de Janeiro RJ Brasil Programa de Pós-Graduação em Medicina Interna, Universidade Federal do Rio de Janeiro - Rio de Janeiro (RJ), Brasil.; 2 Rio de Janeiro RJ Brasil Departamento de Engenharia Industrial, Pontifícia Universidade Católica do Rio de Janeiro - Rio de Janeiro (RJ), Brasil.; 3 Rio de Janeiro RJ Brasil Programa de Pós-Graduação, Instituto D'Or de Pesquisa e Ensino - Rio de Janeiro (RJ), Brasil.

## AO EDITOR

O uso generalizado do aprendizado de máquina criou a possibilidade de gerar modelos de previsão robustos específicos para cada paciente. No entanto, é necessário ter cuidado ao usá-los em populações heterogêneas de pacientes graves.^(1)^ A literatura recente demonstrou grandes avanços no campo da previsão de lesão renal aguda e da necessidade de terapia de substituição renal (TSR).^(2)^ Em uma grande coorte multicêntrica, avaliamos o desempenho de um modelo publicado anteriormente,^(3)^ que prevê a necessidade de TSR em pacientes de unidade de terapia intensiva (UTI) com doença pelo coronavírus 2019 (COVID-19), em pacientes de UTI geral.

Recentemente, usando uma metodologia orientada por dados em uma coorte multicêntrica de 14.374 pacientes graves com COVID-19, desenvolvemos e validamos um modelo de previsão de aprendizado de máquina para prever o uso de TSR (COVID-19-RRT Model).^(3)^ No presente estudo, realizamos uma validação externa do COVID-19-RRT Model em uma coorte de pacientes adultos sem COVID-19 internados em 126 UTIs em 2022 em uma rede hospitalar privada brasileira. Os dados foram adquiridos por meio de uma solução usada para avaliação de qualidade (Epimed Monitor).^(4)^ O estudo foi aprovado pelo Comitê de Revisão Institucional após o fornecimento do consentimento informado (Instituto D'Or de Pesquisa e Ensino [IDOR], CAAE:17079119.7.0000.5249). O desempenho da previsão foi avaliado em termos de calibração (gráficos e pontuação de Brier) e discriminação (área sob a curva ROC [ASC-ROC]). Uma descrição dos materiais e métodos utilizados é apresentada no Material Suplementar (Tabela 1S, 2S e Figura 1S).

Em 2022, 8.735 pacientes de UTI adulto sem COVID-19 precisaram de suporte respiratório precoce. Desses, 770 (8,8%) pacientes foram submetidos à TSR, uma porcentagem menor do que a da coorte de desenvolvimento (12%) (Tabela 1). Os pacientes da coorte de validação externa sem COVID-19 eram mais velhos (idade média de 72 anos *versus* 56 anos), mais frequentemente do sexo feminino (54% *versus* 36%) e mais frequentemente frágeis (43% *versus* 16%) do que os da coorte de desenvolvimento do modelo. A internação média na UTI foi mais curta (6 *versus* 10 dias), e a mortalidade na UTI foi menor em comparação com o grupo de desenvolvimento (18% *versus* 22%). Na coorte não relacionada à COVID-19, a ASC-ROC do modelo foi de 0,82 (intervalo de confiança de 95% [IC95%] 0,80 - 0,83), maior do que na coorte de validação interna (0,79; IC95% 0,78 - 0,82). O escore de Brier foi comparável entre o conjunto de dados de validação externa e o conjunto de dados de validação de intervalo; no entanto, os gráficos de calibração mostraram superestimação das probabilidades de TSR previstas, especialmente para pacientes de baixo risco (Figura 1).

**Figura 1:** Resultados de validação externa da calibração e discriminação do modelo final.

O conjunto de dados de validação externa incluiu 8.735 pacientes com internações em unidades de terapia intensiva geral que precisaram de suporte respiratório na admissão (primeira hora), dos quais 770 precisaram de terapia de substituição renal em 2022. (A) A discriminação foi avaliada usando *receiver operating characteristic* curves e área sob a curva. (B) A faixa de calibração avalia a concordância entre os valores previstos e observados: as áreas sombreadas representam as faixas de confiança (faixa) de 80 e 95%, e a linha vermelha é a linha bissetriz (observado = previsto). Quando as faixas de confiança se desviam significativamente da linha bissetriz, os quantis correspondentes de subestimação ou superestimação são visualizados na parte inferior direita dos gráficos. O valor de p representa um teste estatístico para a hipótese nula de calibração.

**Tabela 1:** Características clínicas e desfechos de pacientes graves da unidade de terapia intensiva geral que precisaram de suporte respiratório (nas primeiras 24 horas após a admissão) e que receberam terapia de substituição renal

Característica			n	Geral n = 8.735	Não TSR n = 7.965	TSR n = 770
Idade			8.735	72 (56 - 84)	72 (56 - 84)	71 (59 - 82)
Sexo			8.735			
	Feminino			4.680 (54)	4.331 (54)	349 (45)
	Masculino			4.055 (46)	3.634 (46)	421 (55)
Índice de Comorbidade de Charlson			8.659	1,00 (0,00 - 3,00)	1,00 (0,00 - 3,00)	3,00 (1,00 - 4,00)
Índice de fragilidade modificado			8.735	2,00 (1,00 - 3,00)	2,00 (1,00 - 3,00)	2,00 (1,00 - 4,00)
Nível do índice de fragilidade modificado			8.735			
	Frágil			3.491 (40)	3.119 (39)	372 (48)
	Não frágil			1.508 (17)	1.435 (18)	73 (9,5)
	Pré-frágil			3.736 (43)	3.411 (43)	325 (42)
Origem da admissão			8.735			
	Pronto-socorro			5.429 (62)	4.936 (62)	493 (64)
	Outra unidade em seu hospital			743 (8,5)	631 (7,9)	112 (15)
	Outros			1.714 (20)	1.632 (20)	82 (11)
	Transferência de outro hospital			324 (3,7)	283 (3,6)	41 (5,3)
	Ala/andar			525 (6,0)	483 (6,1)	42 (5,5)
SAPS-3			8.735	54 (44 - 65)	54 (43 - 64)	65 (55 - 78)
SOFA			6.823	2,0 (1,0 - 5,0)	2,0 (1,0 - 5,0)	7,0 (4,0 - 11,0)
Comorbidades			8.735			
	Hipertensão		8.735	5.364 (61)	4.805 (60)	559 (73)
	Diabetes		8.735	2.909 (33)	2.584 (32)	325 (42)
	Obesidade		8.735	508 (5,8)	468 (5,9)	40 (5,2)
	Imunossupressão		8.735	2.011 (23)	1.810 (23)	201 (26)
	Doença cardiovascular		8.735	2.998 (34)	2.661 (33)	337 (44)
	DPOC ou asma		8.735	1.510 (17)	1.385 (17)	125 (16)
	Malignidade		8.735	1.741 (20)	1.563 (20)	178 (23)
	Doença cerebrovascular		8.735	1.651 (19)	1.547 (19)	104 (14)
	Doença renal crônica		8.735	1.021 (12)	749 (9,4)	272 (35)
	Histórico de tabagismo		8.735	606 (6,9)	558 (7,0)	48 (6,2)
	Cirrose hepática		8.735	197 (2,3)	158 (2,0)	39 (5,1)
	Outras comorbidades		8.735	3.616 (41)	3.247 (41)	369 (48)
Achados fisiológicos na primeira hora						
	Escala de coma de Glasgow mais baixa (1 hora)		8.735	15,0 (11,0 - 15,0)	15,0 (12,0 - 15,0)	14,0 (6,0 - 15,0)
	Contagem de plaquetas mais baixa (1 hora)		8.735	214 (161 - 274)	215 (163 - 275)	192 (130 - 263)
	Ureia		8.735	44 (31 - 67)	42 (30 - 63)	75 (47 - 112)
	BUN		8.735	21 (14 - 31)	20 (14 - 29)	35 (22 - 52)
	Creatinina mais alta (1 hora)		8.735	0,94 (0,70 -1,36)	0,90 (0,70 -1,27)	1,80 (1,10 - 3,20)
Suporte na admissão (1 hora)						
	Ventilação não invasiva		8.735	4.867 (56)	4.575 (57)	292 (38)
	Ventilação mecânica		8.735	2.300 (26)	1.981 (25)	319 (41)
	Vasopressor		8.735	2.099 (24)	1.747 (22)	352 (46)
Suporte durante a hospitalização						
	Suporte de ventilação não invasiva		8.735	6.385 (73)	5.941 (75)	444 (58)
	Cânula nasal de alto fluxo		8.735	247 (2,8)	212 (2,7)	35 (4,5)
	Ventilação mecânica		8.735	3.130 (36)	2.582 (32)	548 (71)
		Dias em ventilação mecânica	3.130	4 (1 - 12)	3 (1 - 10)	9 (3 - 18)
	ECMO		8.735	25 (0,3)	8 (0,1)	17 (2,2)
	Vasopressores		8.735	3.213 (37)	2.625 (33)	588 (76)
Desfechos						
	Mortalidade na UTI		8.735	1.607 (18)	1.244 (16)	363 (47)
	Mortalidade intra-hospitalar		8.735	2.064 (24)	1.623 (20)	441 (57)
	Tempo de internação na UTI		8.735	6 (3 - 13)	6 (3 - 11)	14 (6 - 29)
	Tempo de hospitalização		8.735	10 (5 - 20)	10 (5 - 19)	18 (8 - 38)

TSR - terapia de substituição renal; SAPS-3 - *Simplified Acute Physiology Score III*; SOFA - *Sequential Sepsis-related Organ Failure Assessment*; DPOC - doença pulmonar obstrutiva crônica; BUN - nitrogênio da ureia sanguínea; ECMO - oxigenação por membrana extracorpórea; UTI - unidade de terapia intensiva. Resultados expressos por medianas (intervalos interquartis) ou n (%).

Apesar da boa discriminação, o COVID-19-RRT Model superestimou a probabilidade de necessidade de TSR, especialmente nos estratos de "baixo risco".^(5)^ Isso pode ser explicado pelas diferenças na gravidade da doença basal entre os pacientes com COVID-19 e os pacientes da UTI geral: aqueles tinham uma gravidade basal mais baixa, mas a proporção de uso de TSR foi maior do que a dos pacientes da UTI geral. Por outro lado, um paciente de UTI geral com baixa gravidade basal da doença raramente precisará de TSR. Portanto, apesar do bom desempenho geral, esse modelo tem uso clínico limitado numa população de UTI mista. Nosso estudo reforça a necessidade de modelos com melhor generalização de previsão de TSR e lesão renal aguda em populações mistas de UTI. Além disso, esses achados devem ser interpretados com cautela ao traduzir o uso de modelos desenvolvidos numa população específica num grupo geral de pacientes graves.

## Data Availability

Os dados que fundamentam os resultados deste estudo estão disponíveis com o autor correspondente mediante solicitação razoável.
